# Neutrophils and Immunity: From Bactericidal Action to Being Conquered

**DOI:** 10.1155/2017/9671604

**Published:** 2017-02-19

**Authors:** Tie-Shan Teng, Ai-ling Ji, Xin-Ying Ji, Yan-Zhang Li

**Affiliations:** ^1^School of Medical Sciences, College of Medicine, Henan University, Kaifeng, Henan 475004, China; ^2^Nanshi Hospital, Henan University College of Medicine, Nanyang, Henan 453003, China

## Abstract

The neutrophil is the major phagocyte and the final effector cell of the innate immunity, with a primary role in the clearance of extracellular pathogens. Using the broad array of cytokines, extracellular traps, and effector molecules as the humoral arm, neutrophils play a crucial role in the host defense against pathogen infections. On the other hand, the pathogen has the capacity to overcome neutrophil-mediated host defense to establish infection causing human disease. Pathogens, such as* S. aureus*, have the potential to thwart neutrophil chemotaxis and phagocytosis and thereby succeed in evading killing by neutrophils. Furthermore,* S. aureus* surviving within neutrophils promotes neutrophil cytolysis, resulting in the release of host-derived molecules that promote local inflammation. Here, we provide a detailed overview of the mechanisms by which neutrophils kill the extracellular pathogens and how pathogens evade neutrophils degradation. This review will provide insights that might be useful for the development of novel therapies against infections caused by antibiotic resistant pathogens.

## 1. Introduction

The immune system protects the body from microbes that invade and harm the host. In humans roughly 100 billion neutrophils enter and leave circulating blood every day [1] and constitute the dominant leukocyte population in the circulation, mediate the earliest innate immune responses to infection, and play a pivotal role in the resolution of microbial infections. Neutropenia, an acquired or inherited neutropenia, and neutrophil malfunction result in recurrent, life-threatening infections with bacteria [2].

Neutrophils originate and mature in the bone marrow and are subsequently released into the peripheral vasculature. After a pathogen has breached the epithelial barriers, neutrophils are the first innate immune cells that are rapidly recruited from the bloodstream to sites of infection. Pathogens entry and replication in host tissues lead to the release of exogenous products, such as formyl peptides, lipoproteins, or peptidoglycan. Moreover, the invasive pathogen can also damage body tissues that produce inflammatory signals, for example, chemoattractants and cytokines [3]. These pathogenic products and inflammatory signals are detected by neutrophils via Toll-like receptors (TLRs), G protein-coupled receptors (GPCR), and cognate immune receptors. By sensing the receptor signal, neutrophils will respond to these stimuli, extravasate from blood vessels, and migrate towards the site of infection to phagocytose pathogens. This multistep process encompasses rolling adhesion of neutrophils on endothelial cells, firm adhesion of neutrophils, extravasation through the endothelium, chemotactic migration, and subsequent killing of invading bacterial pathogens. Following migration to the site of infection and phagocytosis, neutrophils have a repertoire of antimicrobial arsenal at their disposal to fulfil this function [4]. Neutrophils utilize a combination of NADPH oxidase-derived reactive oxygen species (ROS), cytotoxic granule components, antimicrobial peptides, and neutrophil extracellular traps (NETs) to generate a highly lethal environment that is essential for efficient microbe killing and degradation [5, 6].

On the other hand, many pathogens have evolved efficient strategies to outfox the weaponry of neutrophils. The main strategies can be divided into five categories: evading extravasation and chemotaxis, preventing opsonization and phagocytosis, surviving inside the neutrophil, inducing cell death, and avoiding killing in NETs [7, 8]. In this review, we will highlight the suite of mechanisms employed by neutrophils to clear bacterial infections and the corresponding counterattack mounted by bacterial pathogens.

## 2. Neutrophil-Mediated Phagocytosis of Pathogenic Microorganism

Initial elimination of invading pathogenic microorganism from human tissue is mediated by professional phagocytes. For efficient phagocytosis, neutrophils first need to leave the bloodstream and reach the site of infection, termed neutrophil recruitment. Furthermore, initiation of phagocytosis requires decoration of bacteria with opsonins that are recognized by specific surface receptors, of which process is termed opsonization of microbes. Lastly, neutrophils express numerous receptors that recognize microbe via binding its specific molecules and host proteins (such as IgG and complement), termed pathogen recognition.

### 2.1. Neutrophils Migrate from the Bloodstream to the Site of Infection

Upon the breach of epithelium by pathogens, as the first responder to microbial invasion, neutrophils leave the bloodstream and move to the site of infection. This recruitment process consists of three major steps: initiation of adherence to activated endothelial cells and rolling, neutrophil arrest caused by firm attachment to the endothelium, and finally migrating across the endothelial barrier to the infection site.

**Figure 1:**
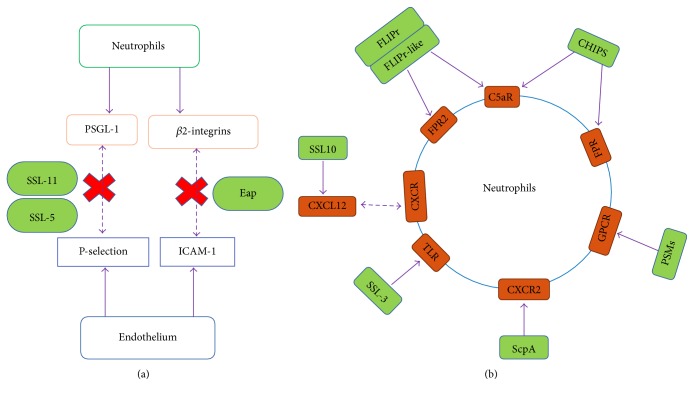
Evasion of neutrophil adhesion and transmigration. (a) Mechanisms by which* Staphylococcus aureus* subverts neutrophil extravasation. (b) Neutrophil attack and evasion of activation.

The initial step occurs through the interaction between the glycoprotein P-selectin glycoprotein ligand-1 (PSGL-1) of neutrophils and P-selectin/E-selectin of endothelial cells [9] (Figure 1(a)). Owing to this loose adhesion, neutrophils can roll along the endothelial cells. The second step is dependent on the interaction between *β*2 integrins (such as LFA-1 and Mac-1) present on the surface of neutrophils and intercellular adhesion molecule 1 (ICAM-1) present on endothelial cells (Figure 1(a)). The final step is triggered by chemokines released by host cells and bacterial products. Host-derived chemokine, such as IL8, GRO-*α*, granulocyte chemotactic protein 2, and complement component C5a/C3a, are potent proinflammatory mediators that are used to recruit additional neutrophils to areas of infection. Furthermore, neutrophils migration also can be elicited by bacteria-derived chemokine, such as lipoteichoic acid or N-formyl peptides (fMLP).

### 2.2. Neutrophil Phagocytosis Is Dependent on Opsonization of Microbes

**Figure 2:**
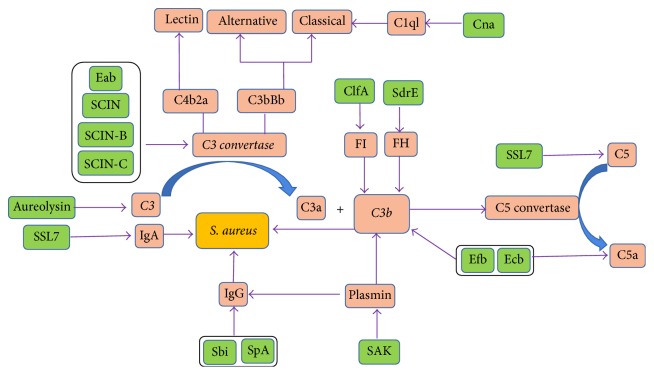
*Staphylococcus aureus* was interfered with chemotaxis and activation of complement.

Initiation of neutrophil phagocytosis is dependent on opsonization of the target microbes that are recognized by specific surface receptors of neutrophils. Complement components and immunoglobulins (Igs) are the predominant factor in serum that enables efficient opsonization. The human complement system is composed of more than 30 proteins and is activated by any one of three routes: the classical pathway, the lectin pathway, and the alternative pathway (Figure 2). Complement system uses three independent pathways to distinguish bacteria from host cells and then can rapidly recognize and opsonize bacteria or kill gram-negative bacteria directly by formation of the membrane attack complex [10]. All three pathways converge in the assembly of a C3 convertase, which are enzyme complexes that consist of C4b2a and C3bBb (C4b2a for the classical and lectin pathways and C3bBb for the alternative pathway). The C3 convertase catalyzes the key reaction in complement activation: cleavage of complement protein C3 results in release of anaphylactic agents C3a and C3b. Most of C3b is further processed into iC3b by complement factor H and complement factor I (Figure 2). At high local concentrations of C3b, the C3 convertase is changed into a C5 convertase, which cleaves C5, resulting in release of the potent chemoattractants C5a, and C5b, which initiates the lytic pathway when deposited on gram-negative bacteria, thereby amplifying the opsonization process.

Igs, which are the second most abundant protein in serum/plasma, play an important role in opsonization of bacteria and subsequent recognition by specific Fc receptor present on the surface of neutrophils. Several Ig subtypes (IgG, IgM, and IgA) have roles in microbial infection control (Figure 2). Different subclasses of Igs display distinct differences in complement activation and Fc*γ* receptor (Fc*γ*R). Normal human neutrophils express two major Fc*γ*Rs, Fc*γ*RII and Fc*γ*RIIIB, and do not express the Fc*γ*R1 [11]. IgG can activate the classical complement pathway and neutralize toxins or other bacterial virulence factors. IgM, owing to its polymeric nature, is particularly effective at complement activation and opsonization. In contrast to IgG and IgM, IgA does not activate the complement system.

### 2.3. Receptor-Mediated Pathogen Recognition and Phagocytosis

Once neutrophils migrate to the site of infection, the opsonized pathogen can be recognized and phagocytized via receptor-mediated uptake into a vacuole within the cell. Similar to other phagocytes, such as macrophages, neutrophils express a large number of receptors including pattern-recognition receptors (PRRs), G protein-coupled receptors (GPCRs), and opsonic receptors. These receptors can recognize microbe-associated molecular patterns (MAMPs) and host proteins (such as IgG and complement) which were used to opsonize the microbe (Figure 1(b)). The PRRs can recognize pathogen-associated molecular patterns (PAMPs), such as bacterial DNA, lipopolysaccharide, peptidoglycan, and lipoteichoic acids. The major types of PRRs on neutrophils include Dectin-1 (recognizing fungal *β*-glucan), triggering receptor expressed on myeloid cells-1 (TREM-1, recognizing bacteria and fungi) [12], and toll-like receptors (TLRs, recognizing lipids, carbohydrates, and peptide). GPCRs, which are expressed in the surface of neutrophils, can recognize bacterial products as well as endogenous molecules released during inflammation. The formyl peptide receptors 1 (FPR1) and its homologue FPR2 belong to the GPCRs family, recognize N-formylated proteins and peptides (fMLP), and consequently induce and potentiate chemotaxis, phagocytosis, and the generation of oxidative burst in neutrophils (Figure 1(b)). Invasive bacterial pathogens are opsonized with complement (e.g., C3b) and antibody (e.g., IgG), recognized by opsonic receptors, including Fc*γ*Rs and the complement receptors (CRs), respectively. Activation of opsonic receptors rapidly enhances the efficiency of phagocytosis and is critical for neutrophil-mediated pathogen killing [13].

## 3. Pathogen Killing by Neutrophils

Neutrophils are the first line of innate immune cells arriving at the site of bacterial inoculation, where they exert diverse antimicrobial mechanisms to prevent pathogen dissemination to normally sterile sites. The process by which neutrophils kill invading pathogens depends on three primary mechanisms [14]: production of highly toxic reactive oxygen species (ROS) in the pathogen-containing vacuole; fusion of neutrophil granules, containing various antimicrobial mediators to the vacuole; NETs formation. These steps may also contribute to inflammatory diseases in which ligands are deposited on tissue components.

### 3.1. Phagocytic Uptake of Bacteria Triggers Production of ROS

Coincident with phagocytosis of bacteria, neutrophils produce an oxidative burst resulting in the rapid release of high levels of bactericidal reactive chemical species under the catalyzation of NADPH oxidase, myeloperoxidase (MPO), or nitric oxide (NO) synthetase [15]. NADPH oxidase is responsible for the generation of ROS, such as superoxide anion (O_2_^−^), hydrogen peroxide (H_2_O_2_), and hydroxyl radicals (HO^*∙*^). The NADPH oxidase functions by shuttling electrons across the phagosomal membrane from cytosolic NADPH to molecular oxygen to produce O_2_^−^. By superoxide dismutase (SOD), the superoxide anion is readily converted to hydrogen peroxide. H_2_O_2_ and O_2_^−^ can combine to generate the highly reactive HO^*∙*^ via the Haber-Weiss reaction, which requires a metal such as iron. As a microbicidal agent, HO^*∙*^ was probably not found in intact cells because that lactoferrin inhibits the generation of HO^*∙*^ and other free radical reactions by binding free copper and iron. Against certain pathogens, such as* Aspergillus*, NADPH oxidase is critical for host defense independently of proteinases, and its importance is revealed in that patients who lack any one of the oxidase subunits suffer from chronic granulomatous disease (CGD) [16].

MPO converts hydrogen peroxide to primarily hypochlorous acid (HOCl). HOCl is the most bactericidal oxidant in neutrophils. Notably, hydrogen peroxide and other secondary oxygen derivatives such hydroxyl radical, chloramines, and HOCl can inactivate iron-sulphur proteins, membrane proteins, and the origin of replication site for DNA synthesis, which play a critical role in the killing of pathogenic bacteria [17]. Indeed, some patients, whose neutrophils lacked MPO, were thought to be immunodeficient [18]. And MPO knockout mice have also shown an undue susceptibility to bacterial and fungal infections [19].

Oxidative deamination of L-arginine by nitric oxide (NO) synthetase generates NO that together with superoxide anion forms reactive nitrogen intermediates with antimicrobial activity [20]. NO, a short-lived (half-life of a few seconds), highly reactive molecule, is produced by inducible nitric oxide synthase (iNOS), which is present in primary granules and is induced upon neutrophil priming (via TNF, IL-1, or IFN-*γ*) and during bacterial infection. NO production complements ROS production by neutrophils to exert antibacterial functions.

### 3.2. Phagocytic Uptake of Bacteria Triggers Production of Degranulation

Pathogens sequestered by neutrophils are trafficked to and fused with the phagosome in a process called degranulation, leading to the killing of invading pathogens in a process involving the release and action of proteinases and peptidases (Table 1). Functionally, the granules can be subdivided into three different classes based on the contents of their matrices and their integral membrane proteins: azurophilic granule, specific granule, and gelatinase granule. Neutrophils are “prepacked” with multiple types of granules that fuse with phagocytic vacuoles to facilitate pathogens destruction. Moreover, granules also help to initiate an inflammatory response and contain alkaline phosphatase, lactoferrin, lysozyme, and NADPH oxidase.

Azurophil (or primary) granules are the first to be produced and contain MPO and a spectrum of neutrophil serine proteases (NSPs): cathepsin G (CG), neutrophil elastase (NE), proteinase 3 (PR3), and the recently discovered neutrophil serine protease-4 (NSP4) [30, 90]. NSPs are critical for the effective functioning of neutrophils and greatly contribute to immune protection against bacterial infections [27]. NSPs are currently believed to have three functions. (1) NSPs can directly kill bacterial cell. NE has been shown to directly kill the gram-negative bacteria* E. coil* by cleavage of its outer membrane protein A, resulting in loss of membrane integrity and cell death. In vivo, the concerted action of NE, CG, and PR3 can kill* S. pneumonia* within phagocytic vacuole. (2) NSPs can cleave host proteins to generate antimicrobial peptides. The best-known example is that PR3 that has been shown to cleave hCAP-18 to generate the antimicrobial peptide LL-37. (3) NSPs can attenuate bacterial virulence by inactivating factors required for pathogenesis.* Shigella flexneri* mobility proteins IcsA and IpaA-C can be cleaved by NE, consequentially preventing its dissemination into the cytoplasm of neutrophils. Similar to NE, CG can cleave the* S. aureus* adhesin clumping factor A and remove its active domain. Together, these NSPs are critical for the effective functioning of neutrophils and immune protection against bacterial infections [27].

In addition, neutrophils also contain a full-length cationic antimicrobial protein, bactericidal/permeability-increasing protein (BPI) in azurophil granules [91]. BPI possesses three types of anti-infective activities: direct antimicrobial activity, neutralizing endotoxin activity through direct binding of LPS, and opsonic activity. BPI binding to LPS results in increased bacterial permeability, hydrolysis of bacterial phospholipids, and death of the bacterium. In addition to its well-documented anti-infective properties, BPI has also been shown to possess additional bioactivities, such as accelerating apoptosis, binding the vascular endothelial growth factor (VEGF), and inhibiting migration of human umbilical vein endothelial cells.

The specific granules are smaller with 0.1 *μ*m diameter and formed after azurophilic granules. These granules do not contain MPO and are characterized by the presence of the glycoprotein lactoferrin. They primarily contain a wide range of antimicrobial compounds including calprotectin, lactoferrin, neutrophil gelatinase-associated lipocalin (NGAL), hCAP-18, and lysozymes. Calprotectin, also called S100A, is a critical factor in the innate immune response to infection and has been shown to inhibit microbial growth through chelation of nutrient Mn^2+^ and Zn^2+^, resulting in reprogramming of the bacterial transcriptome [92]. Lactoferrin, also called lactotransferrin, is an iron-binding glycoprotein present in most biological fluids of mammals and is released from neutrophil granules during inflammatory responses [93, 94]. Lactoferrin possesses a number of types of antibacterial activities: (1) blocking the entry of bacterial pathogens competitively binding onto cell receptors, such as glycosaminoglycans; (2) degrading protein virulence produced by bacteria, such as* H. influenza* and* E. coil*, through proteolysis; (3) preventing bacterial adhesion through competing bacterial adhesion sites on bacteria and host cells [95].

The tertiary granules, also named gelatinase granules, are smaller than specific granules and are both MPO- and lactoferrin-negative. These granules contain few antimicrobials but serve as a storage location for a number of metalloproteases, such as gelatinase and leukolysin. These granules may represent one end of the population of granules formed during neutrophil maturation.

### 3.3. Neutrophil Extracellular Traps Killing Bacteria

In addition to pathogens phagocytosis and subsequent reactive species- and enzyme-dependent pathogen destruction, neutrophils also exert antibacterial activity through neutrophil extracellular traps (NETs), which was first described by Brinkmann et al. in 2004 [96]. Sensing the entry of bacteria, neutrophils extrude a mesh-like structure consisting of DNA/histones and are peppered with granule-derived antimicrobial peptides and enzymes, a process termed NETosis. NETs are composed of DNA strands associated with histones and decorated with about 20 different proteins, including NE, CG, PR3, MPO, lactoferrin, pentraxin 3 [40], high mobility group protein B1, LL37, and buforin II [97]. Mitochondria can also serve as a source of DNA for NET formation. The NETs are capable of ensnaring microbes by localizing and trapping pathogens within a sticky meshwork of chromatin. Furthermore, NETs facilitate pathogen exposing to highly concentrate antimicrobial peptides and enzymes, such as MPO, neutrophil elastase, LL-37, S100A, and lactoferrin-chelating proteins [98]. Along with the chromatin network, these antimicrobial agents are concentrated and the potential for synergistic action is enhanced. When neutrophils extrude a meshwork of chromatin to form NETs, it is not an end point for neutrophils and anuclear neutrophils can also migrate and retain the necessary components to kill bacteria through phagocytosis and formation of mature phagosomes [99].

The molecular mechanisms details of NETs formation are tightly linked to the production of ROS. The magnitude and duration of ROS production play an important role in promoting NETs formation and may be a major role in determining the fate of the neutrophil. In addition, individuals lacking MPO and NADPH oxidase, two key enzymes in the ROS cascade, are unable to make NETs and suffer from debilitating infections [100]. However, ROS are not the only vital roles in NETs formation and decondensation of chromatin is also critical for proper NETs formation. Neutrophil elastase was shown to partially degrade histones and further leads to decondensation of chromatin, which is also a pivotal event in the process of NETs formation [101].

NETosis also has the dark side: apart from this antimicrobial function, the cytotoxicity of NETs can be harmful to the host if their release is inappropriately controlled. Excessive NETs formation is linked to various neutrophil-mediated pathologies, including vasculitis, sepsis, and systemic lupus erythematosus nephritis. NETs also induce platelet procoagulant activation, which can lead to significant thrombosis and vascular injury. Excessive NETs formation and endothelial cell activation are also associated with preeclampsia of pregnancy [102].

## 4. “Catch Me If You Can”: How Pathogens Evade Antibacterial Arsenal of Destruction by Neutrophils

**Figure 3:**
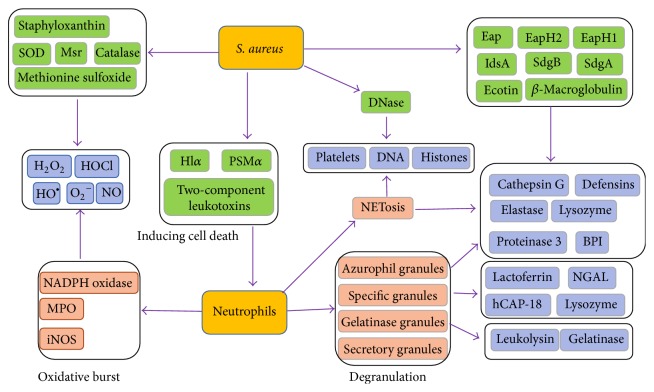
Direct antimicrobial mechanisms from neutrophils and the* S. aureus* counterattack. Neutrophils are equipped with multiple anti-infective strategies including the bacterial uptake (phagocytosis), the phagolysosomal degradation of bacteria via reactive oxygen species (oxidative burst), the release of antimicrobial molecules (degranulation), and the formation of a web-like structure composed of chromatin, histones, and antimicrobials (neutrophil extracellular traps, NETs).* S. aureus* is equipped with a magnitude of neutrophil resistance factors (green boxes) allowing the pathogen to uniquely counteract each antibacterial strategy of neutrophils.

To promote its own survival within the host, bacterial pathogens have evolved an array of specific mechanisms to overcome destructions by neutrophils (Figure 3, Table 2).* S. aureus*, the culprit of many types of infections, exhibits many characteristics of antineutrophils pathogens [103].

### 4.1. Inhibition of Neutrophil Recruitment

Counter measures adopted by pathogen may affect these steps to inhibit neutrophil recruitment. For instance, staphylococcal superantigen-like 5 (SSL5) can block neutrophil adhesion to endothelial cells by binding to PSGL-1 and consequently blocking its interaction with the natural ligand P-selectin [104]. SSL5 and other family members also inhibit leukocyte responses to chemokines, such as CXC, CC, CX3C, and CXCL12, and to the complement fragments C3a and C5a. Moreover, extracellular adherence protein (Eap) generated by* S. aureus* can bind and inhibit ICAM-1, a crucial molecule used to facilitate the neutrophils firm adhesion of endothelial cells. Furthermore, bacteria can secrete a variety of proteases, leading to degradation of chemokines. Chemotaxis inhibiting protein (CHIPS), a protein freely secreted by* S. aureus*, binds directly to the C5a receptor and formyl peptide receptors (FPRs) and thereby inhibits neutrophils recruitment [105, 106]. As a homologue of CHIPS in* S. aureus*, FPR-like 1 inhibitory proteins (FLIPr and FLIPr-like) bind and inhibit FPR1 as well as C5aR and then impair neutrophil chemotaxis. Another cysteine protease secreted by* S. aureus* is staphopain A, which inactivates CXCR2 chemokines by cleaving its N-terminal domain and then inhibits neutrophil activation and recruitment [53]. In addition, SSL3 specifically binds and inhibits TLR2 activation, which is critical for host defense against* S. aureus* [107].

### 4.2. Preventing Phagocytosis

*S. aureus* has successfully developed ways to evade the complement system by secretion of specific complement inhibitors (Figure 2, Table 2). The secreted factors described below allow bacteria to either diminish or delay the detrimental effects of an innate immune attack, thereby generating a window of opportunity to replicate and establish a microenvironment conducive to bacterial survival and disease pathogenesis [108, 109].

#### 4.2.1. Cleavage of IgG

SSL7 binds host IgA and complement component C5, inhibiting generation of C5a, phagocytosis, and production of phagocyte reactive oxygen species.* S. aureus* expresses two surface-anchored proteins, staphylococcal protein A (SpA) and staphylococcal immunoglobulin-binding protein (Sbi), which impair IgG function. SpA possesses five immunoglobulin-binding repeat domains. Each domain can bind the Fc-part of IgG, thereby blocking the interaction with Fc receptors on neutrophils. Sbi consists of four small domains, of which two (Sbi-I and Sbi-II) can bind IgG [110–112].

#### 4.2.2. Direct Inactivation of C3 Convertases

It has been shown that SCIN and its homologues (SCIN–B and SCIN–C), as strongly antiphagocytic molecules, modulate all the three complement pathways through the unique interaction with C3 convertases [61]. Extracellular fibrinogen-binding protein (Efb) and its homologue extracellular complement-binding protein (Ecb) can modulate the alternative pathway convertase by binding to the C3b molecule directly [113].* S. aureus* secretes the 16 Kda Efb that binds two different plasma proteins using separate domains: the Efb N-terminus binds to fibrinogen, while the C-terminus binds complement C3b.

#### 4.2.3. Binding or Cleavage of Human Convertase Regulators

*S. aureus* recruits the complement regulatory protein factor H (fH) and factor I (fI) to its surface to inhibit the alternative pathway of complement activation. The surface-associated protein SdrE, as an fH-binding protein, enhances recruitment of fH which resulted in increased iC3b generation [73]. The clumping factor A (ClfA) of* S. aureus* binds to complement regulator factor I and increases factor I cleavage of C3b [114–116]. Similar to ClfA, iron-regulated surface determinant protein H (IsdH) could act as a factor I-mimicking protease and directly trap factor I to the* S. aureus* surface, promoting cleavage of C3b [74].

#### 4.2.4. Eliminating Opsonic Molecules from the Bacterial Surface

Staphylokinase (SAK) is a secreted protein that binds and activates surface-bound plasminogen into plasmin, which removes IgG as well as C3b from the bacterial surface, making this protein a unique antiopsonic molecule [67]. Aureolysin [60], a secreted metalloprotease, inhibits the deposition of C3b on* S. aureus* surfaces and the release of the chemoattractant C5a. It has been shown that aureolysin cleaves the central complement protein C3 specifically in the *α*-chain, close to the C3 convertase cleavage site, yielding active C3a and C3b. The antiphagocytic activity of the capsule is well established and the quantity of capsule is decisive for* S. aureus* and virulence. Overexpression of capsular polysaccharides type 8 renders* S. aureus* more resistant to phagocytosis by neutrophils in vitro [72, 117].

### 4.3. Surviving inside the Neutrophil

The combined action of ROS and antimicrobial proteins generated by granules creates a lethal environment for microbes. However,* S. aureus* harbored by neutrophils can survive in the presence of extreme environment, although not replication [118]. This is because* S. aureus* has evolved many means to resist oxidant damage and antimicrobial proteins degradation, as well as surviving within phagosomes.

First of all,* S. aureus* strains can express five types of enzymes or pigment promoting resistance to oxidative killing by stimulated neutrophils, including superoxide dismutase, catalase, staphyloxanthin, methionine sulfoxide reductases (Msr), and adenosine synthase A (AdsA). Superoxide dismutase produced by* S. aureus* can convert superoxide anion to H_2_O_2_, which is then consumed to yield O_2_ and H_2_O by catalase, thereby eliminating oxidants generated by stimulated neutrophils. Furthermore,* S. aureus* strains also produce the pigment staphyloxanthin, which consumes oxidants and renders bacteria resistant to oxidant-dependent killing, protecting bacteria from singlet oxygen via an undefined mechanism. Msr is a highly conserved enzyme that repairs oxidative damage incurred within neutrophils, contributing to survival of bacteria within neutrophils. AdsA, a cell wall-anchored enzyme, can convert adenosine monophosphate to adenosine [119]. As a critical virulence factor, AdsA promotes staphylococcal synthesis of adenosine in blood, escaping from phagocytic clearance [84]. Adenosine is also known to inhibit neutrophil degranulation, adhesion to vascular surfaces, and superoxide burst [120]. These findings indicate that phagocytosed* S. aureus* devote significant energy and effort to self-preservation rather than to growth and replication.

Bacteria pathogens have evolved two strategies to counteract human NSPs. The first one is modifications of bacterial NSPs substrates. For gram-positives, such as* S. epidermidis* and* S. aureus*, glycosyltransferases (SdgA and SdgB) are expressed to modify the serine-aspartate dipeptide repeats (SDR) of GLcNAc, which will protect these bacteria from proteolytic degradation by CG. The LPS of Gram-negatives, such as* Neisseria meningitidis*, are anchored to the outer membrane by lipid A. The lipid can be modified by phosphoethanolamine transferase to prevent proteolysis-independent killing by CG.

The second strategy is production of NSPs inhibitors. A recent study reports that* S. aureus* has evolved three highly specific NSPs inhibitors: extracellular adherence protein (Eap) and its smaller homologues EapH1 and EapH2 [88]. These proteins are very potent and specific inhibitors of NSPs and imply a crucial role for NSPs in the defense against* S. aureus*. Iron-regulated surface determinant protein H (IsdH) is present in the surface of* S. aureus*, which binds to lactoferrin, the most abundant antistaphylococcal polypeptide [121]. IsdA confers resistance to killing by lactoferrin. In addition, recombinant IsdA was a competitive inhibitor of lactoferrin protease activity. Thus, IsdA can protect* S. aureus* against lactoferrin and acts as a protease inhibitor [122].

### 4.4. Inducing Cell Death by Cytolytic Toxins

Following phagocytosis of bacteria pathogens, neutrophils would kill most bacteria and have initial features typical of apoptosis. However,* S. aureus* can survive within these neutrophils and ultimately cause cytolysis. Recent studies have provided evidence that cytolytic toxins produced by* S. aureus* contribute to neutrophil lysis after phagocytosis [123]. Cytolytic toxins produced by* S. aureus*, including the phenol soluble modulins (PSMs), alpha-hemolysin (Hl*α*), and two-component leukotoxins, facilitate neutrophil killing after phagocytosis [124]. PSMs were first identified in 1999 by hot phenol extraction from* S. epidermidis* culture filtrate, in which three peptides termed PSM*α*, PSM*β*, and PSM*γ* were identified. PSMs do not have uniform charge characteristics. PSM*α*s of* S. aureus* are positively charged, while PSM*β* peptides are all negatively charged, and the PSM*γ* is neutral [125]. In* S. aureus*, PSM*α* peptides have a pronounced ability to lyse human neutrophils, in which PSM*α*3 has by far the strongest activity. However, PSM*γ* (also named *δ*-toxin) has moderate cytolytic activity and the PSM*β* peptides are noncytolytic. At the micromolar concentrations, PSM*α* has the pronounced capacity to kill human neutrophils after phagocytosis by disrupting the cytoplasmic membrane [126]. While at nanomolar concentrations, PSM*α* may stimulate neutrophils and initiate proinflammatory responses including neutrophil chemoattraction, activation, and the release of IL-8 [127]. Neutrophils sense PSMs via formyl peptide receptor 2 (FPR2), which may sense the amphipathic, *α*-helical structure of PSMs rather than a specific amino acid sequence motif.

Panton-Valentine Leukocidin (PVL) is a prophage-encoded pore-forming exotoxin, which mainly acts on neutrophils as a crucial virulence factor in necrotizing diseases. PVL is a staphylococcal bicomponent pore-forming toxin comprising the protein subunits LukS-PV and LukF-PV [128]. Initial binding of LukS-PV to the surface of target cells triggers secondary binding of LukF-PV and subsequently induces the assembly of lytic pore-forming [129]. PVL-induced pore formation is mediated by the human C5aR, which determines species specificity of PVL [79]. The C5aR can bind LukS-PV, which is a potent inhibitor of C5a-induced immune cell activation.

*S. aureusα*-hemolysin (*α*-toxin, Hla) belongs to the class of small *β*-barrel pore-forming cytotoxins [130]. As a water soluble monomer, *α*-hemolysin is capable of binding and oligomerization into a heptameric structure on neutrophils. Then, *α*-hemolysin exhibits the main action on pore formation and neutrophils lysis after phagocytosis [131]. In other studies, *α*-hemolysin has been suggested to directly disrupt the* S. aureus* phagosome and promote* S. aureus* escape to and replication in the cytoplasm [131].* S. aureusα*-hemolysin facilitates the secretion of newly synthesized CXC chemokines into the airway and stimulates neutrophil homing in staphylococcus aureus pneumonia [132].

### 4.5. Avoiding Killing in NETs

In addition to phagocytosis and intracellular killing, neutrophils release NETs that capture and kill microbes in the extracellular space. Several bacterial pathogens have evolved sophisticated mechanisms to suppress, escape, and/or resist NETs. Expression of nucleases is one highly conserved anti-NET factor among bacteria, which can degrade NETs indicating that the chromatin functions as a scaffold and is a major component of the fibres. Interestingly, extracellular nucleases are found in several pathogenic bacteria including* S. aureus*,* Clostridium perfringens*, and* S. pyogenes* (group A* Streptococcus*, GAS) [133].

In addition to produce nucleases, GAS can also suppress NETs formation by degrading the neutrophil stimulatory chemokine IL-8 with peptidase SpyCEP or HA capsule engagement of the inhibitory neutrophil receptor Siglec-9. Other GAS resistance factors, including M1 protein, Scl-1 protein, and the GLcNAc side chain, contribute to GAS resistance to antimicrobial components.

## 5. Conclusion

The interaction between neutrophils and pathogens remains a fascinating subject. The host requires the action of neutrophils to fight invaders and the pathogens in turn must cope with neutrophil attacks in order to colonize the host [134]. Several pharmacological agents can be used to enhance neutrophil energy generation, antimicrobial activities, and treatment outcomes. For instance, hypoxia-inducible factor 1 (HIF-1), innate defense regulator peptides (IDRs), and vitamin B3 all enhance antimicrobial activities to provide prophylactic and therapeutic activity against bacterial pathogens in vivo. And tamoxifen [135] or anacardic acid [136] could boost NETs formation and bacterial killing of neutrophils. To overcome antibiotic resistant pathogens which harness the multifaceted antimicrobial properties of neutrophils, these host-directed strategies provide a critical new element to boost neutrophil function and minimize the risk for development of antibiotic resistance during infection.

## Figures and Tables

**Table 1:** Mechanism of action of neutrophil antimicrobial proteins/peptide.

Antimicrobial protein/peptide	Direct antimicrobial mechanism	Alternative antimicrobial mechanism	Subcellular localization	Ref.
*α*-Defensins	Membrane-active; inhibition of DNA, RNA, protein, bacterial cell wall synthesis	Opsonisation of bacteria/ROS formation	Primary granules, NETs	[21]
LL-37	Transmembrane pore-forming	ROS formation	Secondary granules, NETs	[22]
BPI	Hydrolysis of bacterial phospholipids by binding to LPS	Inhibiting cytokine liberation by binding to CD14	Primary granules	[23, 24]
Histones	Membrane-active	NETs formation	Nucleus, NETs	[25]
Lysozyme	Degrades bacterial cell wall	NETs formation	Lysosomes	[5, 26]
PR3	Proteolytic activity; degrading virulence factors	NETs formation	Primary granules/NETs	[27–29]
NE	Proteolytic activity; degrading virulence factors	NETs formation	Primary granules/NETs	[27–29]
CatG	Proteolytic activity	NETs formation; ROS formation	Primary granules/NETs	[28, 29]
NSP4	Trypsin-like activity	Unknown	Primary granules	[30–32]
Azurocidin	Membrane-active	Opsonisation of bacteria	Primary granules	[33]
Lactoferrin	Altering bacterial growth by binding to iron; increase in membrane permeability by binding to the lipid A	Decreasing the release IL-1, IL-2, and TNF*α*; Suppressing NETs release	Secondary granules/NETs	[34–37]
Calprotectin	Altering bacterial growth by sequestering Mn^2+^ and Zn^2+^	Inhibition of Mn^2+^-dependent bacterial superoxide defenses; NETs formation	Secondary granules	[38, 39]
PTX3	As a soluble pattern recognition receptor in innate immunity	NETs formation	Secondary granules/NETs	[40]
NADPH oxidase	Generation of superoxide anion	NETs formation	Lysosomes	[41]
MPO	Generation of hypochlorous acid	NETs formation	Lysosomes	[18, 42, 43]
Platelets	Activating neutrophils to release NETs	NETs formation	NETs	[44]
NGAL	Inhibit bacteria growth by capturing and depleting siderophores	Acting as a growth and differentiation factor in multiple cell type	Secondary granules	[45, 46]

**Table 2:** Neutrophil antibacterial functions subverted by *S. aureus*. *S. aureus* produces a large suite of virulence factors to counteract specific neutrophil clearance mechanisms during the pathogenesis of invasive infection.

Virulence factor	Targets	Function	Ref.
SSL-5	PSGL1/GPCRs	Recruitment/chemotaxis inhibition	[47, 48]
SSl-6	PSGL1	Recruitment inhibition	[49]
SSl-11	PSGL1	Recruitment inhibition	[50]
SSl-3	TLR2	Chemotaxis inhibition	[51, 52]
SEIX	PSSG1	Recruitment inhibition	[49]
ScpA	CXCR2	Chemotaxis inhibition	[53]
CHIPS	FPR1, C5aR	Chemotaxis inhibition	[54, 55]
FLIPr	FPR2	Chemotaxis inhibition	[56, 57]
FLIPrL	FPR1, FPR2	Chemotaxis inhibition	[56, 57]
PSMs	FPR2	Chemotaxis inhibition/neutrophils lysis	[56, 58]
Eap	ICAM1/C4b/NE/CG/PR3	Recruitment/phagocytic inhibition	[59]
Aureolysin	C3	Complement inhibition	[60]
SCIN	C3bBb	Complement inhibition	[61, 62]
SCIN-B/C	C3bBb	Complement inhibition	[61, 62]
Efb	C3b	Complement inhibition	[63]
Ecb	C3b	Complement inhibition	[64]
SSL7	IgA/C5	Phagocytosis/complement inhibition	[65]
SSL10	IgG	Phagocytosis inhibition	[66]
SAK	C3/IgG	Phagocytosis inhibition	[67]
Sbi	IgG/C3/factor H	Phagocytosis inhibition	[68, 69]
SpA	IgG	Phagocytosis inhibition	[70]
ClfA	Factor I	Phagocytosis inhibition	[56]
SOK	Unknown	Phagocytosis inhibition	[71]
CP	Unknown	Phagocytosis inhibition	[72]
SdrE	Factor H	Complement inhibition	[73]
IsdH	C3b	Complement inhibition	[74]
Cna	C1q	Complement inhibition	[75]
LukAB	*α*M integrin	Neutrophils lysis	[76]
LukED	CCR5/CXCR1/CXCR2	Neutrophils lysis	[77]
LukMF	Not known	Neutrophils lysis	[78]
PVL	C5aR	Neutrophils lysis	[79]
Hla	C5aR	Neutrophils lysis	[80]
Staphyloxanthin	Unknown	Resistance to ROS	[81]
KatA	Hydrogen peroxide	Resistance to ROS	[82]
AhpC	Hydrogen peroxide	Resistance to ROS	[82]
Msr	Hydrogen peroxide	Resistance to ROS	[83]
AdsA	Adenosine	Resistance to ROS	[84]
IsdA	Fibrinogen	Resistance to lactoferrin	[85]
OatA	Peptidoglycan	Resistance to lysozyme	[86, 87]
EapH1	NSPs	Resistance to NSPs	[88]
EapH2	NSPs	Resistance to NSPs	[88]
Nuclease	DNA	Resistance to NETs	[89]
